# Oncocytic intraductal papillary neoplasm of the pancreas: Multimodality imaging and cytopathologic correlation

**DOI:** 10.4102/sajr.v30i1.3390

**Published:** 2026-05-20

**Authors:** Anju Ranga, Pranav Gupta, Agrima Bansal, Ravindra K. Saran, Sudeshdeep Sinha, Kalpana Bansal

**Affiliations:** 1 Department of Radiology, Govind Ballabh Pant Hospital, New Delhi, India

**Keywords:** intraductal oncocytic papillary neoplasm, IOPN, pancreatic cystic neoplasm, MRI pancreas, mural nodule

## Abstract

Intraductal oncocytic papillary neoplasm of the pancreas (IOPN-P) is a rare cystic epithelial tumour with distinctive imaging, cytological and histological features. This case report describes a 65-year-old female who presented with abdominal pain and vomiting for 6 months and was later diagnosed with IOPN-P. Multimodality imaging, including ultrasound, contrast-enhanced CT, MRI, MRCP and endoscopic ultrasound, played an important role in lesion evaluation. Cytopathological evaluation and immunohistochemistry confirmed the diagnosis.

**Contribution:**

The case highlights the importance of recognising radiological features of IOPN-P to differentiate it from more aggressive pancreatic neoplasms.

## Introduction

Intraductal oncocytic papillary neoplasm of the pancreas (IOPN-P) is a rare intraductal epithelial tumour, characterised by complex papillary architecture and oncocytic cytoplasm.^1,2^ Although previously classified as a subtype of intraductal papillary mucinous neoplasm (IPMN), it has been recognised as a distinct entity in the 2019 WHO Classification of Digestive System Tumours related to its unique morphological, immunohistochemical and molecular features.^1,2^ As a result of its rarity, detailed imaging descriptions remain limited in the literature. This report describes a case of histologically confirmed IOPN-P with detailed multimodality imaging and cytopathologic correlation, highlighting key distinguishing features from other pancreatic cystic lesions.

## Case presentation

A 65-year-old female presented with complaints of abdominal pain and episodes of vomiting, persisting for 6 months. Laboratory blood investigations were unremarkable.

Ultrasound of the abdomen (Figure 1) revealed a well-defined cystic lesion in the distal body of the pancreas with few thin internal septa and a hyperechoic solid nodule, without significant vascular flow on colour Doppler. Contrast-enhanced CT abdomen (Figure 2) confirmed a hypodense cystic lesion in the distal body of the pancreas measuring 4.7 cm × 4.8 cm × 4.1 cm (AP × TR × CC). Few thin enhancing septations were seen within. A solid mural nodule was observed along the posterior aspect, with enhancement similar to the adjacent pancreatic parenchyma on the pancreatic and portal venous phases (isoenhancing). The rest of the pancreas was normal in bulk and enhancement, with no dilatation of the main pancreatic duct (MPD).

**FIGURE 1:**
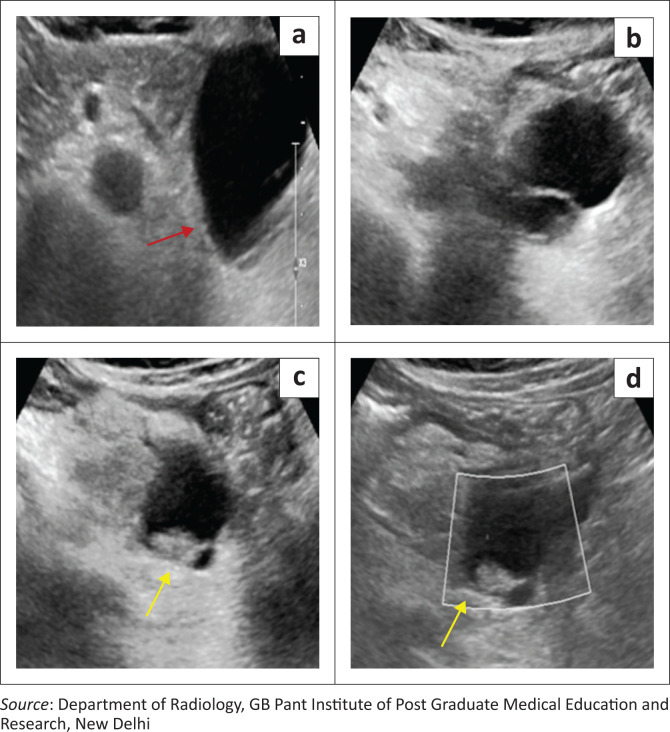
(a–d): Axial ultrasound images of the pancreas reveal a well-defined, rounded, anechoic cystic lesion (red arrow) in the distal body of the pancreas with few, thin, internal septa and a solid appearing, hyperechoic nodule (yellow arrow) without any vascularity on colour doppler imaging (d).

**FIGURE 2:**
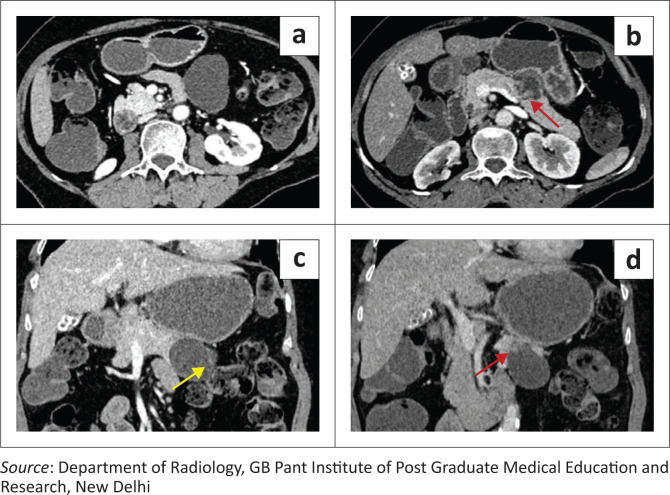
Axial (a, b) and coronal (c, d) contrast-enhanced CT images of the abdomen demonstrate a hypodense, cystic lesion in the distal pancreatic body with thin, enhancing internal septa (yellow arrow). An isoenhancing solid nodule is noted within the lesion, displaying enhancement similar to the adjacent pancreatic parenchyma (b, d; red arrows). The remaining pancreas appears normal in size, attenuation and enhancement, with no significant dilatation of the main pancreatic duct.

MRCP (Figure 3) demonstrated a well-defined, round, T1 hypointense, T2 Spectral Attenuated Inversion Recovery (SPAIR) hyperintense, cystic lesion with few T2 hypointense internal septations, 1.3 mm thick. The mural nodule appeared isointense on T1, iso- to hypointense on T2 and demonstrated restricted diffusion on DWI. No restriction was present in the cystic component. No obvious MPD communication or upstream dilatation of the MPD was seen.

**FIGURE 3:**
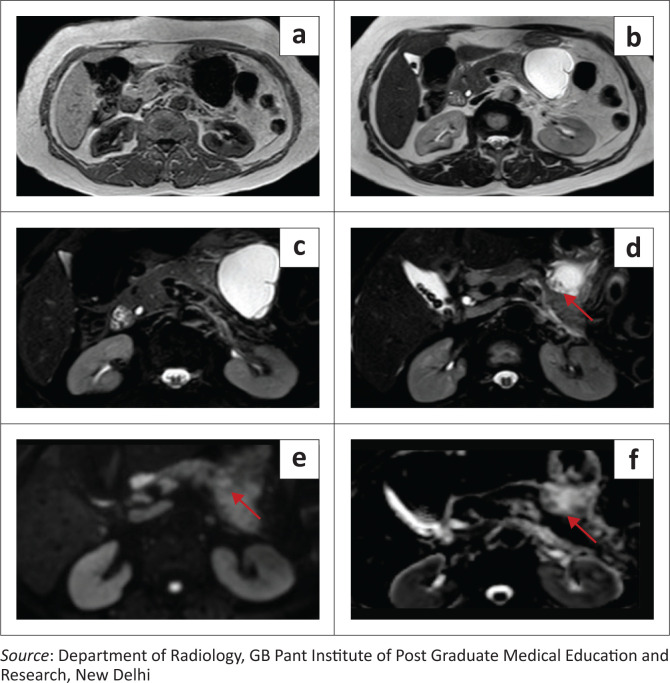
(a, b) Axial MRI images of the abdomen demonstrate a cystic lesion that appears hypointense on T1-weighted imaging (a) and hyperintense on T2-weighted imaging (b). (c, d) A nodule is seen along the inferior aspect of the lesion (red arrows), isointense on both T1- and T2-weighted images. DWI (e) and ADC map (f) show diffusion restriction within the solid nodule (red arrows), with no diffusion restriction in the cystic component.

Subsequent endoscopic ultrasound (EUS) demonstrated a 6 × 4.7 cm cystic lesion in the body of the pancreas with thin septations and a single 6 mm mural nodule. The lesion appeared to be communicating with the MPD, which appeared mildly prominent (~3 mm) distal to the lesion. Thick fluid was aspirated from the lesion, and the string sign was positive. Fine needle aspiration cytology (FNAC) of the mural nodule was performed. Cytosmears were moderately cellular, showing atypical cells in sheets, clusters, papillaroid fragments and small groups (Figure 4). Focal acinar formation was observed. The cells exhibited overlapping, nuclear crowding, moderate pleomorphism, hyperchromatic round to oval nuclei, prominent nucleoli and moderate eosinophilic cytoplasm. Few cells had intracellular mucin. The background showed mucin admixed with haemorrhage. Cell block showed tumour cells in clusters with gland formation, moderate pleomorphism, vesicular chromatin, prominent nucleoli and moderate eosinophilic cytoplasm. Intracellular mucin was seen in some cells. On immunohistochemistry (IHC), tumour cells were strongly positive for cytokeratin 19 (CK19) and pan-cytokeratin (PanCK). Chromogranin and insulinoma-associated protein 1 (INSM1) were negative. Endoscopic ultrasound-guided FNAC findings were suggestive of adenocarcinoma.

**FIGURE 4:**
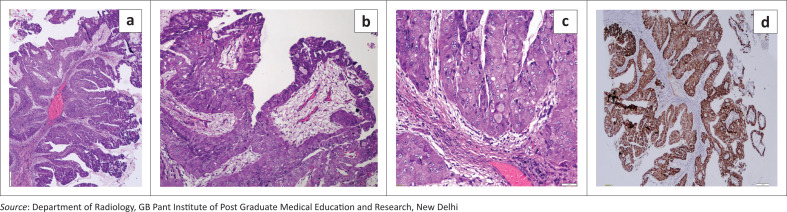
(a–d) (a) Low-power view showing papillary fronds lined by tumour cells with abundant eosinophilic cytoplasm (hematoxylin and eosin [H&E] stain). (b) Higher magnification highlighting the vesicular nuclei of oncocytic tumour cells. (c, d) Immunohistochemistry demonstrates strong positivity for Hepatocyte Paraffin 1 (HepPar-1) and MUC5AC in the tumour cells, supporting the diagnosis of intraductal oncocytic papillary neoplasm (IOPN).

The patient underwent surgical excision of the lesion. On gross examination, sectioning of the cystic lesion revealed mucinous fluid. On histopathology, a cystic lesion was seen with a papillary growth measuring 2 × 1 × 0.5 cm. The cut surface was grey-white. The distance of the growth from the stapled margin was 0.5 cm. The microscopy section showed a cystic tumour composed of tumour cells arranged in complex arborising papillae and a cribriform pattern. The tumour cells were round with abundant granular oncocytic cytoplasm, round nuclei with coarse clumped chromatin, and prominent nucleoli. The cribriform structures contained mucin. Interspersed goblet cells were seen. On IHC, these cells were positive for HepPar, Mucin 5AC (MUC5AC) and negative for mucin 2 (MUC2) (Figure 4). A final diagnosis of IOPN-P was made.

## Discussion

Although initially grouped under IPMNs, IOPN-P was reclassified as a separate entity in 2019 because of distinct histopathological and molecular features.^1,2^ Unlike conventional IPMN subtypes (gastric, intestinal and pancreatobiliary), IOPN-P exhibits significantly lower frequencies of KRAS, GNAS, and and RNF43 mutations.^3,4^ Instead, it frequently has unique gene fusions such as ATP1B1-PRKACB, DNAJB1-PRKACA and ATP1B1-PRKACA, which are not observed in other pancreatic or biliary neoplasms.^3^

Morphologically, IOPN-P tends to form intricate masses with both solid and cystic components and produces minimal mucin, often mimicking pancreatic ductal adenocarcinoma on imaging.^4,5,6^ Despite its distinct pathological profile, data on the radiologic presentation of IOPN-P remain scarce.^4,6^ On cross-sectional imaging, particularly contrast-enhanced CT and MRI, IOPNs often present as large, multilocular cystic masses with internal solid components or mural nodules.^4,5,6,7^ These lesions frequently involve the MPD, leading to its segmental or diffuse dilatation.^4,6^ However, unlike other IPMN subtypes, IOPNs tend to produce less mucin, resulting in less pronounced downstream MPD dilatation.^4,5^ MRI typically shows these lesions as hyperintense on T2-weighted images and hypointense on T1-weighted sequences, with post-contrast enhancement of nodular or papillary structures and septae.^5,6,7^ Flurodeoxyglucose PET may additionally reveal intense radiotracer uptake because of the high mitochondrial content in oncocytic cells, although this metabolic activity does not necessarily correlate with aggressive behaviour.^2,4,6^ The presence of large solid components, relatively preserved surrounding tissue planes, and low incidence of invasive features on imaging support the diagnosis of IOPN and may help it to distinguish it from more aggressive neoplasms such as pancreatic ductal adenocarcinoma.^4,5,6,7^

The lesion in this case appeared cystic with thin septations and a mural nodule, which was solid on ultrasound and showed enhancement on CT, similar to pancreatic parenchyma.^4,5,6^ On MRI, the lesion exhibited hypointense signal on T1-weighted images, hyperintense signal on T2-weighted images.^5,6,7^ The mural nodule was isointense and showed diffusion restriction on DWI sequences, indicative of high cellularity.^4,5,6^ Although MRCP did not demonstrate a definite communication with the MPD, EUS suggested subtle communication. This discrepancy may be attributed to the higher spatial resolution of EUS and the possibility of detection of even a small ductal communication that may not be appreciable on MRCP.

Differential considerations for cystic pancreatic lesions with mural nodules include IPMN, mucinous cystic neoplasm (MCN), as well as cystic pancreatic neuroendocrine tumour (cNET)(Table 1). Characteristic communication with the pancreatic ductal system is seen with IPMNs, which often demonstrate significant MPD dilatation with abundant mucin production.^4,5,6^ The absence of significant MPD dilatation and lack of pathognomonic mucin hypersecretion typically associated with the ‘fish-mouth’ ampulla, allowed for the exclusion of IPMN in the current case.^4,5,6^ In contrast, IOPNs typically produce less mucin and may show only mild or segmental ductal dilatation; although ductal communication can be present, it may be subtle or not visualised on MRCP as in this case.^4,5,6^ A MCN was ruled out based on clinical and morphological discordance. Unlike the complex intraductal growth observed in this case, MCNs typically present as thick-walled, multiloculated cysts with septations in the pancreatic body or tail that lack communication with the ductal system and are histologically defined by the presence of ovarian-type stroma.^5,6^ In addition, the prominent enhancing mural nodules identified in the patient are atypical of MCN. Cystic pancreatic neuroendocrine tumours may show cystic degeneration but are typically hypervascular lesions with avid arterial phase enhancement and lack intraductal growth or papillary architecture.^6^ In this case, the mural nodules demonstrated isoenhancement relative to the pancreatic parenchyma, a feature more consistent with IOPN. Additional distinguishing features of IOPN include the presence of complex papillary architecture, relatively preserved surrounding pancreatic parenchyma and lower incidence of invasive features despite the often large lesion size.^4,5,6^ Recognition of these imaging characteristics, in conjunction with clinical and cytopathologic findings, is essential for differentiating IOPN from other cystic pancreatic neoplasms and avoiding misdiagnosis. Cytological findings in this case were consistent with oncocytic differentiation – cells with abundant eosinophilic cytoplasm, nuclear pleomorphism and occasional mucin production.^1,2,8^ Immunohistochemistry confirmed epithelial origin (CK19 and PanCK positive) and excluded neuroendocrine differentiation.^2,8^

**TABLE 1:** Key imaging features differentiating intraductal oncocytic papillary neoplasm from intraductal papillary mucinous neoplasm, mucinous cystic neoplasm and cystic pancreatic neuroendocrine tumour.

Feature	IOPN	IPMN	MCN	cNET
Duct communication	Often present (may be subtle)	Characteristic	Absent	Absent
MPD dilatation	Mild/segmental	Marked	Absent	Absent
Mucin production	Minimal	Abundant	Present	Absent
Morphology	Cystic with solid papillary components	Ductal dilatation ± nodules	Thick-walled cyst	Solid tumour with cystic degeneration
Mural nodules	Common	May be present	May be present	Less typical
Enhancement	Isoenhancing mural nodules	Variable	Wall/septa enhancement	Hyperenhancing

IOPN, intraductal oncocytic papillary neoplasm; IPMN, intraductal papillary mucinous neoplasm; MCN, mucinous cystic neoplasm; cNET, cystic pancreatic neuroendocrine tumour; MPD, main pancreatic duct.

Endoscopic ultrasound-guided FNAC in this case was initially interpreted as suggestive of adenocarcinoma. This highlights the limitation of FNAC interpretation, as cytological and nuclear atypia may fulfil the criteria for malignancy, while invasion cannot be assessed. Assessment for invasion is only possible on histopathology or may be suggested on high-resolution imaging.^2,8,9^ This represents a known diagnostic pitfall.^9^ Typically, IOPNs demonstrate significant cytologic atypia, including nuclear pleomorphism, prominent nucleoli and architectural complexity, which may overlap with features of ductal adenocarcinoma.^2,8^ Recognition of oncocytic cytoplasm as a soft pointer, along with correlation with imaging findings, may help to avoid misinterpretation as adenocarcinoma.^4,5,6^ Although IOPN often shows high-grade dysplasia, it tends to have low invasive potential and a favourable prognosis compared to intraductal papillary mucinous carcinoma or pancreatic ductal adenocarcinoma.^1,2,10,11^ Recognition of its distinct imaging and pathological profile is crucial for appropriate diagnosis and surgical planning.^4,5,6^

## Conclusion

Intraductal oncocytic papillary neoplasm of the pancreas is a rare pancreatic neoplasm with distinct imaging and cytological features. Multimodality imaging, especially the identification of mural nodules and septations, is vital for early detection and differentiation from more aggressive pancreatic malignancies. Cytopathology, histopathology and IHC remain the mainstay for confirmation of diagnosis and guide appropriate management.
